# Evaluation of the Antimicrobial Properties of a Natural Peptide from *Vespa mandarinia* Venom and Its Synthetic Analogues as a Possible Route to Defeat Drug-Resistant Microbes

**DOI:** 10.3390/biology11091263

**Published:** 2022-08-25

**Authors:** Jin Zhang, Ruize Sun, Zhiwei Chen, Chunyuan Zhou, Chengbang Ma, Mei Zhou, Xiaoling Chen, Tianbao Chen, Chris Shaw, Lei Wang

**Affiliations:** Natural Drug Discovery Group, School of Pharmacy, Queen’s University Belfast, 97 Lisburn Road, Belfast BT9 7BL, UK

**Keywords:** wasp venom, antimicrobial peptides, peptide modification, *Galleria mellonella* larva model

## Abstract

**Simple Summary:**

With the prevalence of antimicrobial resistance caused by drug abuse, severe infection currently remains an urgent global challenge. The antimicrobial peptides rich in wasp venom advance the development of new drugs to addressing this issue. A natural peptide from wasp venom was discovered with a great antimicrobial activity against Gram-positive bacteria both in vitro and in vivo. Meantime, by adapting for specific substitution of amino acid, the engineered peptide exhibited more potency and broader spectrum of antimicrobial activity against Gram-positive bacteria, Gram-negative bacteria and yeast.

**Abstract:**

Antimicrobial peptides (AMPs) from wasp venom have a good track record and potential for drug development as tools against development of antimicrobial resistance. Herein, the biological function and activity profile of peptide VM, which was discovered in the venom of the wasp, *Vespa* *mandarinia*, and several of its third-position substituted analogues, were investigated. VM had potent antimicrobial activity against Gram-positive bacteria and biofilm, and all modified peptides achieved the significant enhancement of these capacities. The various physicochemical properties of amino acids substituted in analogues, generated the different mechanisms of action of bacterial membrane disruption. VM-3K showed a maximum 8-fold enhancement of antibacterial activity against Gram-positive bacteria and also presented microbicidal properties against Gram-negative bacteria and fungi. This peptide also exhibited a high killing efficiency at low concentration and had a comparable selectivity index to VM. Furthermore, VM-3K produced a 90% survival of *S. aureus*-infected waxworms at a concentration of 5.656 mg/kg, at which concentration the natural template peptide only achieved 50% survival. This peptide also lacked short-term resistance generation. Thus, peptide VM-3K could be a promising broad-spectrum antimicrobial candidate for addressing the current antibiotic-resistant infection crisis. It is worth mentioning that this investigation on the relationship between peptide structure and mechanism of action could become an important aspect of drug research on short peptides.

## 1. Introduction

The worldwide prevalence of antimicrobial resistance (AMR) is now playing an increasingly important role in our daily lives, and it has been recognised as one of the most serious threats to the world, causing a global public health crisis. The broad spread of microbial drug-resistance has made the treatment of common infections increasingly difficult [1]. A systematic study in 2019 illustrated that there were approximately 4.95 million deaths caused by infection worldwide, of which 1.27 million deaths could be directly related to AMR [2]. The World Health Organisation (WHO) have already announced that the antibiotic resistance crisis had become one of the direst catastrophes for humankind. Many countries have already set up policies to respond to this threat, while the exploration for novel alternative products is being taken ever more seriously.

To combat the shortage of pathogen killers, antimicrobial peptides (AMPs) are gaining traction as alternative therapies in the pharmaceutical industry. AMPs are one of the most important categories of active peptides and these include host-defence peptides, which possess cytotoxic activities against bacteria, fungi, viruses and other tissue-invading organisms [3]. Large numbers of AMPs have anti-bacterial functions and can effectively suppress the growth of various clinical pathogens. Many AMPs have inhibitory abilities against both Gram-positive bacteria and Gram-negative bacteria, including *Staphylococcus* spp., methicillin-resistant *Staphylococcus aureus* (MRSA), *Proteus* app., *Enterococcus* spp., *Escherichia coli* (*E. coli*), *Bacillus subtilis*, *Pseudomonas* spp. and others [4], and both ribosomally-synthesised and non-ribosomally-synthesised peptides show antibacterial activities [5]. With the rapid evolution of anti-fungal drug resistance, a generation of new anti-fungal AMPs is also required. Indolicidin, a cationic antimicrobial peptide-amide isolated from cytoplasmic granules of bovine neutrophils, can inhibit fungi by interacting with their target cell lipid bilayer [6]. Two pore-forming peptides, opistoporin-1 and parabutoporin, were also confirmed to have the ability of inhibiting the growth of fungi [7].

In addition to their excellent biological activities, AMPs have a wide scope for research because of their systematic and typical characteristics. AMPs are composed of 10 to 60 amino acids and can form linear or cyclic structures [8]. They have both hydrophobic and hydrophilic regions, and more than half of their amino acids are hydrophobic [9]. To prolong the half-lives and improve the activities of AMPs, post-translational modifications, such as C-terminal α-amidation, act by blocking carboxypeptidase actions and providing a hydrogen bond which can form an α-helix and add a positive charge [10].

Their wide distribution and abundant sources give AMPs a broad acquired space and huge application potential. Peptides are a large component of the defence systems found in nearly all forms of organisms, ranging from microorganisms, plants to vertebrates as well as invertebrate species [11]. Wasp venom is a treasure trove of peptides, which exhibit multiple functions, including antimicrobial, histamine releasing, anti-inflammatory and anticoagulant activities [12]. One of the most famous venom peptides, honeybee melittin, displays better antibacterial activity against Gram-positive bacteria than negative bacteria but also has antifungal activity [13,14]. Another peptide from wasp venom, mastoparan, also exhibits antibacterial activities and its lysine-substituted analogue, (MK58911) and has potent antifungal properties [15,16].

In this paper, an AMP from *Vespa mandarinia* venom, namely VM, was studied with regard to investigating its antimicrobial and antibiofilm properties. The peptide sequence was found in the NCBI database, and very few cited studies have examined this peptide for its antibacterial properties against *Staphylococcus aureus* (*S. aureus*), *E. coli* and *Candida albicans* (*C. albicans*) [17]. The research described here, was to investigate the biological activity of this peptide in more depth, especially with regard to AMR bacteria, along with its mechanism of action. Meanwhile, four third-position substituted analogues were designed and their bio-functions were explored both in vitro and in vivo. Furthermore, their therapeutic capacity was evaluated compared with the template peptide, VM, through research on physicochemical characteristics, the mechanism of action and cytotoxicity. In summary, this article describes an approach for generating antimicrobial peptides through rational design, which have more potent and broader spectrum microbicidal activities with low toxicity in vivo and have potential impact in generating novel antimicrobial mechanisms.

## 2. Materials and Methods

### 2.1. Peptide Design and Synthesis

The sequences of template peptide and modified analogue peptides are shown in Table 1. VM was first identified in the venom of the wasp, *Vespa mandarinia* [18]. Proline in the short, α-helical-structural sequence, normally plays a role of helix breaker [19]. In this study, the third-position proline (from the N-terminus) was replaced by either lysine, glycine, tryptophan or tyrosine, respectively, to produce the analogues VM-3K, VM-3G, VM-3W and VM-3Y in order to investigate the biological activity consequences.

The peptides with amidation were synthesised through solid phase peptide synthesis (SPPS) using a Tribute^®^ 2-Channel Peptide Synthesiser (Protein Technologies, Tucson, AZ, USA) with Rink amide MBHA resin and purified by reverse-phase high performance liquid chromatography (RP-HPLC; Waters, Miford, MA, USA) with an analytical Aeris 5 µm PEPTIDE XB-C18 column (250 mm × 21.2 mm, Phenomenex, Macclesfield, Cheshire, UK). The peptides were identified and confirmed by time-of-flight mass spectrometry (MALDI-TOF MS) on a linear time-of-flight Voyager DE mass spectrometer (Perseptive Biosystems, Foster City, MA, USA). The HPLC chromatograms (Figure S1) of the purified peptides and corresponding mass spectra (Figure S2) were provided in the supplementary document.

### 2.2. The Determination of Peptide Secondary Structures and Physicochemical Characteristics

The secondary structures of peptides were analysed by use of a JASCO J815 circular dichroism (CD) spectrometer (Jasco, Essex, UK). The peptides were prepared in 10 mM NH_4_Ac and 50% (*v*/*v*) trifluoroethanol (TFE)/10 mM NH_4_Ac at a concentration of 100 µM, respectively. The CD spectra were recorded at wavelengths ranging from 190 nm to 250 nm, with a 200 nm/min scan speed, a 1 nm bandwidth and a 0.5 nm data pitch. The CD spectra were plotted by using GraphPad Prism version 6.01 (GraphPad Software, La Jolla, CA, USA) and their structural constitutions were analysed by an online secondary structure determining and peptide-fold recognising software, BeStSel (http://bestsel.elte.hu/) (accessed on 20 December 2021) [20].

The physicochemical properties (hydrophobicity <*H*>), hydrophobic moment <μM> and net charge were predicted by an online software, HeliQuest (http://heliquest.ipmc.cnrs.fr/) (accessed on 20 December 2021) [21].

### 2.3. Antimicrobial Assays

The minimal inhibitory concentration assay (MIC) and minimum bactericidal concentration assay (MBC) of the peptide were carried out using the broth dilution method. Seven microbes, including the Gram-positive bacteria, *S. aureus* (NCTC 6538), MRSA, (ATCC 12493) and *Enterococcus faecalis* (*E. faecalis*) (NCTC 12697); Gram-negative bacteria, *E. coli* (ATCC 8739), *Klebsiella pneumoniae* (*K. pneumoniae*) (ATCC 43816) and *Pseudomonas aeruginosa* (*P. aeruginosa*) (ATCC 9027); and a yeast, *C. albicans* (ATCC 10231), were used in these assays and incubated with peptide at 37 °C overnight. The growth culture media were Yeast Extract Peptone Dextrose for *C. albicans* and Mueller–Hinton broth for others. The concentration of microbes was detected by measuring OD values through the use of a UV spectrophotometer set to 550 nm. *C. albicans* assays required 10^6^ colony forming units (CFU)/mL, while others were incubated with 10^8^ CFU/mL and verified by viable cell counts. All of the inocula were diluted to final concentrations of 5 × 10^5^ CFU/mL. The concentration of peptides ranged from 1 µM to 512 µM (C = 1, 2, 4, 8, 16, 32, 64, 128, 256, 512 µM). Two mg/mL of amphotericin B for *C. albicans* and 2 mg/mL norfloxacin for others were applied as positive controls, and 1% of dimethyl sulphoxide (DMSO) was applied as a vehicle control. The 96-well plates were analysed at 550 nm using an ELISA plate reader (Biolise BioTek EL808, Winooski, VT, USA).

After analysing the growth situation of microbial cultures from each concentration, 10 µL of the inhibited cultures (the peptide concentration ranges from MIC to maximum concentration) were selected to sub-culture onto the Mueller–Hinton agar (MHA) plates. The plates were incubated overnight at 37 °C. After incubation, the lowest concentration on the MHA plates with no bacterial growth was regarded as the MBC value.

### 2.4. Antibiofilm Assays

The minimal biofilm inhibitory concentration assay (MBIC) and minimal biofilm eradication concentration assay (MBEC) were performed on each of the peptides using Gram-positive bacteria cultured in Tryptic Soy Broth (TSB, Sigma-Aldrich, St. Louis, MO, USA) and Gram-negative bacteria cultured in Luria-Bertani broth (LB, Thermo Fisher Scientific, Carlsbad, CA, USA). The seeding concentration in 96-well plates of bacterial suspension was the same as for the anti-bacterial assay, and a UV spectrophotometer set to 550 nm was used to detect OD values. Melittin was used as a positive control. For MBIC, peptide solution was mixed with bacterial suspension at first to test if it could inhibit the growth of biofilm. However, for minimal biofilm eradication (MBEC) assays, biofilm was formed in 96-well plates at 37 °C for 24 h after seeding bacteria; afterwards, it was washed by sterile PBS twice. Peptide solutions, prepared with culture broth, were added to form the biofilms by incubating at 37 °C for 24 h. Both plates were washed by PBS twice and stained by 125 µL 0.1% crystal violet solution (Sigma-Aldrich, Gillingham, UK). The stain was dissolved in 150 µL 30% acetic acid (Sigma-Aldrich, Gillingham, UK). The absorbance values of 100 µL solution from each well, were analysed by using a Synergy HT plate reader (Biotech, Minneapolis, MN, USA) set at 595 nm.

### 2.5. Kinetic Time Killing Assay

This assay was designed to analyse the bactericidal kinetics of VM and modified peptides against *S. aureus*. The concentrations of peptide used corresponded to the MIC value and 2× MIC value. The bacterium was cultured in 100 mL MHB at 37 °C until 10^8^ CFU/mL was reached, as for the standard anti-bacterial assay. After diluting the bacterial suspension to 5 × 10^5^ CFU/mL, the mixture of peptide and bacterial inoculum was incubated at 37 °C. At 0, 10, 20, 30, 60, 90, 120 and 180 min, the bacterial colony counts were tested on MHA plates at 37 °C for 24 h after dilution in PBS.

### 2.6. Membrane Permeabilisation Assay

Bacteria were incubated in TSB at 37 °C overnight, and 200 µL of bacterial suspension was diluted into 25 mL TSB loaded into a 50 mL centrifuge tube to subculture at 37 °C for 2 h. Then the tube with bacteria was centrifuged at 1000× *g* for 10 min at 4 °C and the supernatant was removed. The bacteria at the bottom were washed twice by 5% TSB in 0.85% NaCl solution. Bacteria were suspended in 5% TSB in 0.85% NaCl solution to achieve a 0.7 OD value at 590 nm. The test mixture consisted of 50 µL of bacterial inoculum; 40 µL of peptide solution for the sample and 40 µL of 5% TSB solution for negative control; and 10 µL of 1% SYTOX Green Nucleic Acid Stain (Life Technologies, Carlsbad, CA, USA). The system was incubated in the dark at 37 °C for 2 h and was analysed using an ELISA plate reader (Biolise BioTek EL808, Winooski, VT, USA) with excitation at 485 nm and emission at 528 nm.

### 2.7. Haemolysis Assay

Defibrinated horse erythrocytes (TCS Biosciences Ltd., Buckingham, UK) were used in the haemolysis assay. Peptides, from 1 μM to 512 μM, were tested with a 4% (*v*/*v*) erythrocyte suspension and were incubated at 37 °C for 2 h, while 1% Triton X-100 (Sigma-Aldrich, St. Louis, MO, USA) was acted as a positive control and phosphate-buffered saline (PBS) was used as a negative control. The sample supernatants were analysed at 550 nm on an ELISA plate reader (Biolise BioTek EL808, Winooski, VT, USA).

### 2.8. Efficacy Evaluation against S. aureus In Vivo

The anti-bacterial activities of VM and analogue peptides were assessed in living larvae of *Galleria* *mellonella*. The waxworms (250 ± 25 mg) (Livefood UK Ltd., Rooks Bridge, UK) were infected with 10 µL of *S. aureus* (NCTC 6538) suspension (1 × 10^7^ CFU/mL) for 1 h and were then injected with 10 µL of peptides. The concentrations (mg/kg) of each peptide were calculated from the MIC value, 2× MIC and 4× MIC value (µM), through unit conversion, respectively. Furthermore, 10 µL of PBS and 20 mg/kg vancomycin were injected into separate larvae as negative controls and positive controls, respectively. Ten waxworms were treated in each group and the numbers surviving were counted each 24 h for five days. Additionally, some larvae were treated only with peptide solution at 4× MIC value to evaluate the potential toxicity in vivo.

### 2.9. Resistance Induction by Serial Passage

This assay was used on four peptides to investigate whether they could induce the development of drug resistance [22,23]. Based on the results of anti-microbial assays, the 1/2 MIC value of each peptide was calculated, respectively. *S. aureus* (NCTC 6538) was harvested and incubated with the peptides at concentrations of 1/2 of respective MICs. Afterwards, the treated bacteria were transferred into fresh MHB and utilised for a further anti-microbial assay. The process was repeated for 16 times.

## 3. Results

### 3.1. The Physicochemical Properties of VM and Modified Analogues

The physicochemical properties of five peptides were calculated by HeliQuest (Table 2). VM had more non-polar than polar residues, so it displayed a high hydrophobicity of around 1.0. Among modified analogues, VM-3K had the lowest hydrophobicity, which could be attributed to the polar, cationic nature of lysine. The aromatic amino acid modifications in VM-3W and VM-3Y both showed varying higher hydrophobicity compared to VM. VM-3K and VM-3G displayed increases in hydrophobic moment, which indicated they had higher amphiphilic character than the parent peptide.

### 3.2. Conformational Study by CD

**Figure 1:**
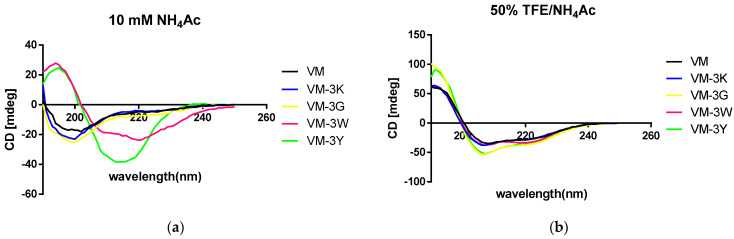
CD spectra recorded for all VM family peptides (100 μM) in (**a**) 10 mM NH_4_Ac; (**b**) 50% (*v*/*v*) trifluoroethanol (TFE)/10 mM NH_4_Ac. Each CD spectrum represents the average of three scans.

In an aqueous environment, unlike the other three peptides, which adopted random coil structures, VM-3W and VM-3Y folded into beta-sheets (Figure 1). Meanwhile, the analogues VM-3G, -3W and -3Y showed higher proportions of α-helix compared to the parent peptide, VM (Table 3).

In a membrane-mimetic environment, the structures of all five peptides were dominated by α-helix (Figure 1). More than 75% of VM-3G and VM-3Y displayed an α-helical structure in 50% (*v*/*v*) TFE/NH_4_Ac solution (Table 3).

### 3.3. Anti-Microbial Activity of VM and Its Analogues

VM displayed typical temporin peptide characteristics, which were a potent anti-bacterial activity against Gram-positive bacteria but limited bactericidal activity against Gram-negative bacteria (Table 4).

With substitutions of the third amino acid of the VM peptide, all analogues displayed stronger bactericidal activities against Gram-positive bacteria and VM-3K exhibited the most potent activity even against conventional drug-resistant bacteria. Meanwhile, VM-3K also showed potent anti-Gram-negative bacterial activity, with a greater than 32-fold increase in potency over VM being observed. Interestingly, VM-3G also displayed anti-microbial ability against Gram-negative bacteria and had bactericidal effects on *E. coli* and *P. aeruginosa* but not on *K. pneumoniae*. Except for VM-3K, the other peptides with third-position substitutions lost their ability to kill yeast.

### 3.4. Anti-Biofilm Activity of VM and Its Analogues

For Gram-positive bacterial biofilm, the parent peptide and analogues displayed potent biofilm inhibitory activities, with inhibitory concentrations either equal to or two-fold higher than their MIC values (Table 5). Among third-position substitutions, VM-3W exhibited weaker biofilm inhibitory abilities against *S. aureus* and MRSA than the other three peptides, while, interestingly, it was more effective than VM-3Y in inhibiting *E. faecalis* biofilm. Additionally, all five of the VM peptides showed eradication activity against *S. aureus* and MRSA.

As in the antimicrobial assays, only VM-3K could inhibit the growth of Gram-negative bacteria below 100 µM and none could eradicate the biofilm from Gram-negative bacteria.

### 3.5. Time-Killing Kinetics of VM and Its Analogues

**Figure 2:**
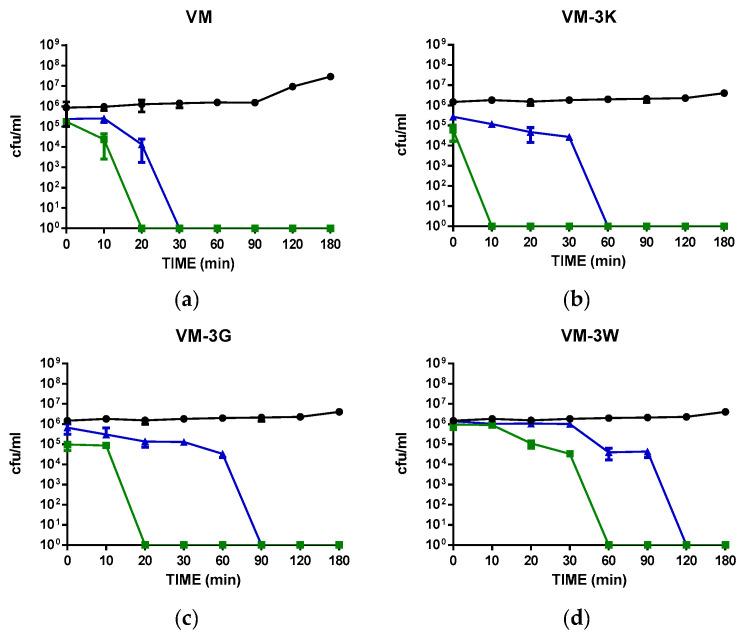

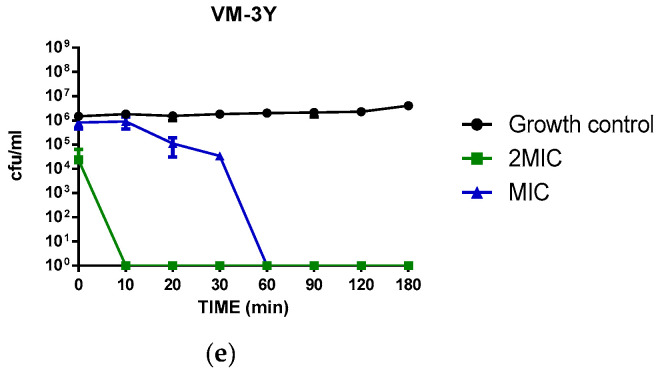
Bacterial killing kinetics of five VM peptides that have potent antibacterial ability against *S. aureus*. (**a**) The bacterial killing kinetics of VM; (**b**) The bacterial killing kinetics of VM-3K; (**c**) The bacterial killing kinetics of VM-3G; (**d**) The bacterial killing kinetics of VM-3W; (**e**) The bacterial killing kinetics of VM-3Y. The bacteria were treated with peptides for 180 min at concentrations corresponding to 1× MIC and 2× MIC. The bacteria treated with broth only were used as growth controls. Data points represent mean ± standard error of means (SEM) of nine replicates from three independent experiments.

To investigate the killing efficiency of five peptides, *S. aureus* was used in these assays (Figure 2). The concentrations of peptides used were 1× MIC and 2× MIC of relevant peptides. All five peptides eradicated *S. aureus* in 2 h at MIC values and at the higher concentration, VM eradicated the bacteria in 30 min. There were three peptides (VM-3K, VM-3G and VM -3W) used at 1 µM and 2 µM. Among these, VM-3K had the best efficiency and could eradicate the bacteria in 10 min at 2 µM. VM-3Y had the same kinetics as VM-3K at 4 µM. At the same concentration (2 µM), VM-3Y and VM-3W displayed similar bactericidal efficiencies after 60 min.

### 3.6. Bacterial Cell Membrane Permeabilisation of VM and Its Analogues

**Figure 3:**
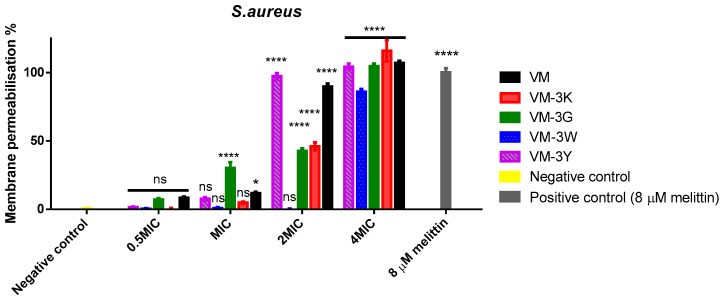
Cell membrane permeabilization effects of peptides against *S. aureus*. Peptide concentrations were 0.5× MIC, 1× MIC, 2× MIC and 4× MIC. Bacteria incubated in relevant broth were treated as the negative control, and 8 µM melittin acted as the positive control. Data points represent mean ± SEM of nine replicates from three independent experiments. The statistical significance was analysed using two-way ANOVA with Dunnett’s multiple comparisons test in GraphPad Prism software by comparison with the negative control. The significance is indicated by asterisks (* *p* < 0.05; **** *p* < 0.0001), and ns represents no significant difference.

To explore the Gram-positive bactericidal mechanism of action of VM and its analogues, *S. aureus* was used in these experiments (Figure 3). For *S. aureus*, most peptides started disturbing membranes from 2× MIC and reached around 100% membrane permeabilization at 4× MIC (Figure 3). Compared with others, VM-3G displayed a much higher membrane-permeable proportion at the concentration of MIC (1 µM), and the parent peptide VM demonstrated a smaller proportion of membrane permeability at its MIC value (4 µM). VM and VM-3Y achieved complete membrane permeabilization (≥90% membrane permeability) at 2× MIC. VM-3G showed a gradual increase in permeabilization with concentration.

### 3.7. Haemolytic Activities of VM and Analogues

**Figure 4:**
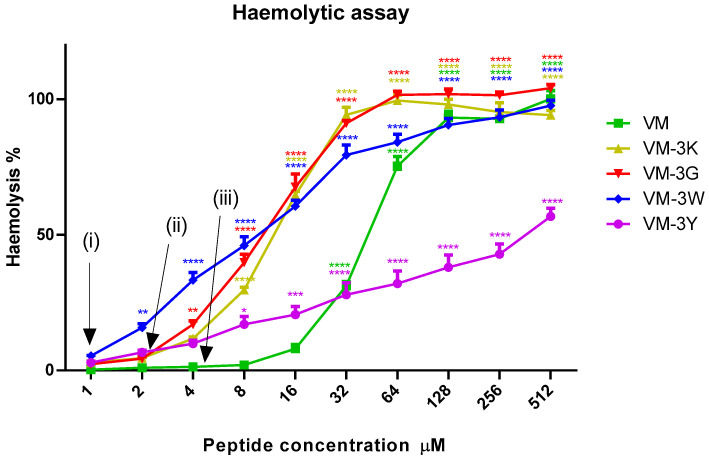
Haemolytic activity of peptides to horse erythrocytes. The haemolytic activities are presented as percentages normalised to a positive control (100% haemolysis caused by 1% Triton X-100). The horse red blood cells incubated with PBS were used as the negative control. Data points represent mean ± SEM of nine replicates from three independent experiments. The statistical significance was analysed using two-way ANOVA with Dunnett’s multiple comparisons test in GraphPad Prism software by comparison with the negative control (* *p* < 0.05, ** *p* < 0.01, *** *p* < 0.001, **** *p* < 0.0001). (i) shows the MIC concentration of VM-3K, VM-3G and VM-3W; (ii) shows the MIC concentration of VM-3Y; and (iii) shows the MIC concentration of VM.

The maximum concentration of VM that caused less than 10% haemolysis was 16 µM, which was four-fold higher than the MIC value against *S. aureus* (Figure 4). All the four modified peptides showed higher haemolysis than the template peptide at 32 µM, but VM-3Y displayed more moderate cell lytic activity at high concentrations, which made its HC_50_ value (the peptide concentration leading to 50% lysis of horse erythrocytes) dramatically low than the other peptides (Table 6). VM-3W and VM-3G had comparable HC_50_ values, but VM-3W exhibited haemolysis at a low concentration. Nevertheless, at the concentrations of their minimum MIC values, the haemolytic capacity of all peptides was very close to 0%. Moreover, though VM-3K caused 50% damage of erythrocytes at 11.2 µM (calculated), it only presented around 10% haemolytic activity at the concentration of 4× MIC (4 µM).

Selectivity index (SI) is a ratio that showed the window between cytotoxicity and anti-microbial ability by dividing a 50% lysis of horse red blood cells into the minimum concentration value of microbial growth inhibition [24]. From Table 6, VM-3Y displayed a low sensitivity towards red blood cells, and with strong anti-bacterial ability (2 µM against *S. aureus* and MRSA), VM-3Y had a significant selectivity index of 105.6. Meanwhile, VM-3G, -3K and -3W had a lower value of HC_50_, but along with a lower value of MIC, they still obtained a comparable SI to the natural peptide.

### 3.8. The Anti-Bacterial Efficiency of VM and Modified Peptides In Vivo

**Figure 5:**
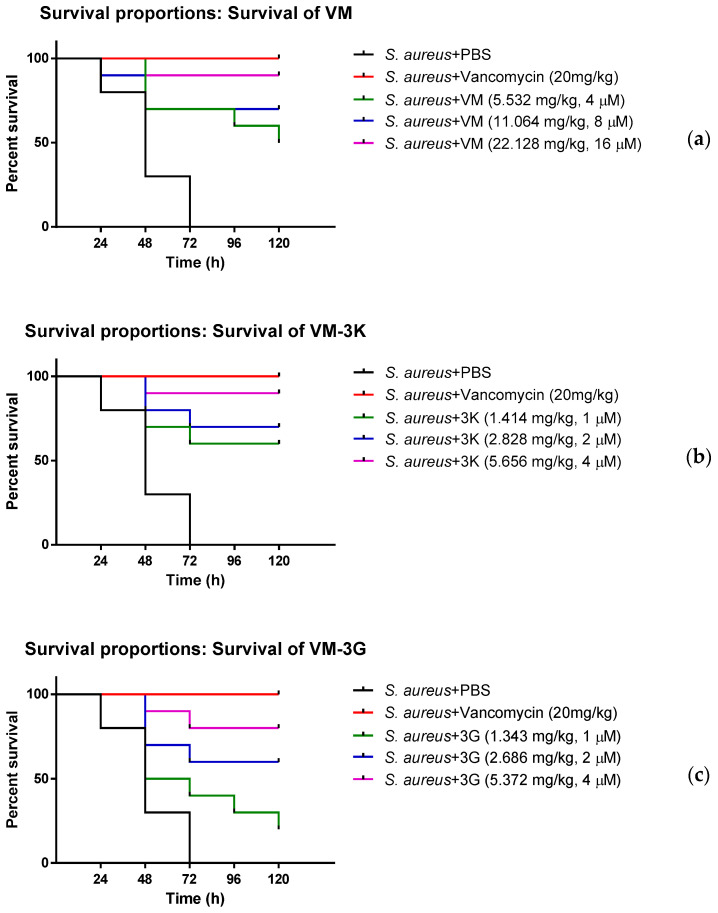

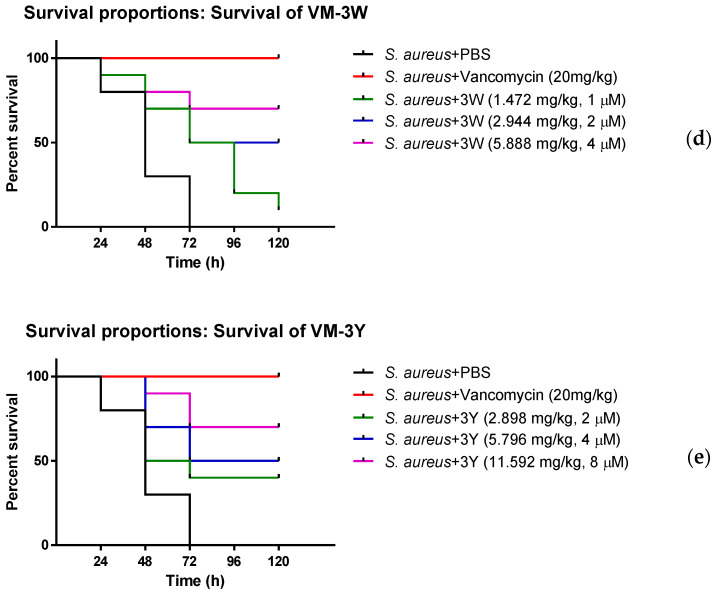
The survival proportions in waxworm larvae (*n* = 10) infected with *S. aureus* and treated with (**a**) VM; (**b**) VM-3K; (**c**) VM-3G; (**d**) VM-3W; and (**e**) VM-3Y. The concentrations (mg/kg) of peptides were converted from their 1× MIC, 2× MIC and 4× MIC values (µM). PBS and 20 mg/kg vancomycin were utilised as negative controls and positive controls, respectively.

VM and modified peptides displayed potent anti-bacterial activities against Gram-positive bacteria in vitro. In this assay, waxworms showed different survival rates after infection with *S. aureus* (Figure 5). The 4× MIC of each peptide in PBS exhibited no observable toxicity to the waxworms even after five days. The treatment with VM resulted in a high survival rate of 90% at 4× MIC (16 µM, 22.128 mg/kg), and it still showed 50% survival at 1× MIC (4 µM, 5.532 mg/kg). For modified peptides, VM-3K performed better than the parent peptide, in which it achieved a 90% survival rate at a concentration of 4 µM (5.656 mg/kg), compared to the 1× MIC of VM (4 µM, 5.532 mg/kg), which achieved just a 50% survival rate. For VM-3G and VM-3W, the peptides produced high survival rates at high concentrations. However, at their lower bactericidal concentrations, they still performed better than VM and displayed 30% (VM-3G) and 20% (VM-3W) higher survival rates at comparable concentrations. It was worth noting that VM-3Y had a two-fold lower MIC value than VM but still had a similar anti-bacterial ability in vivo at a comparable concentration.

### 3.9. Resistance Induction by Serial Passage in S. aureus

**Figure 6:**
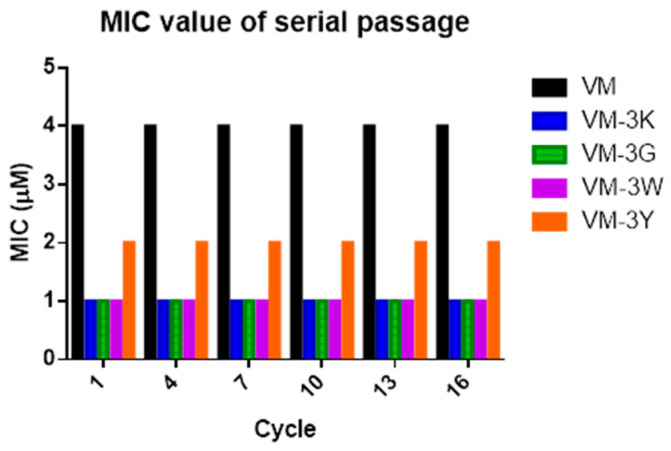
Assessment of the drug-resistance induction of VM and modified peptides in *S. aureus* after 16 passages. The *X*-axis represents the cycles of passage, and the *Y*-axis represents the MIC value of each peptide/analogue used.

After 16 passages of *S. aureus* treated with VM and its analogues, the anti-bacterial activity of each peptide did not show a decrease (Figure 6). This suggested that these five peptides did not induce drug-resistance during the time of testing.

## 4. Discussion

As promising candidate drugs for the drug industry, peptides are known for their diverse functional biological activities and their flexible characteristics for research on composition and structure. In this study, a temporin peptide was chosen as a template to study the relationships between the specific substitution of amino acids and bio-active functions. Temporin peptides (VM) from wasp, *Vespa mandarinia* venom, display typical temporin family characteristics, including the presence of the classical hydrophobic amino acid motif, “FLP-“, at the N-terminus, and amidation at the C-terminus. Due to the high proportion of hydrophobic amino acids, VM had high hydrophobicity values, up to 1.045. Additionally, VM showed high amphiphilicity whose hydrophobic moment was calculated as 0.618. CD spectroscopy illustrated that VM had an α-helical structure, which displayed 64.3% of helicity in membrane-mimetic environments. These typical characteristics led VM to have many of the bio-functions that temporin peptides demonstrate. It displayed potent antimicrobial activities against Gram-positive bacteria (MICs: 4–16 µM) and yeast (MICs: 32 µM) but only displayed limited inhibitory ability against Gram-negative bacteria (MICs: 256–>512 µM). VM also had the ability to inhibit the growth of Gram-positive bacterial biofilm, and to eradicate biofilms of *S. aureus* and MRSA (MBECs: 64 µM). The moderate haemolytic activity of VM showed that it possessed a selectivity between horse red blood cell and microbial cell targets.

The structure–activity relationship of temporin was studied based on the substitution of proline-3 with lysine, glycine, tryptophan and tyrosine in a series of analogues. These third-position proline modifications exhibited a maximum of 8-fold enhancement of anti-bacterial activities against Gram-positive bacteria. The MICs/MBCs of VM-3K, VM-3G and VM-3W against *S. aureus* were as low as 1 µM, and VM-3K eradicated *E. faecalis* at a concentration of 2 µM, which indicated that the replacement of the third-position proline by these four amino acids, was an effective method to enhance the anti-Gram-positive bacterial activities of VM. From the results of kinetic time-killing assays, the efficiency of bactericidal activity of third-position proline analogues was, VM-3K > VM-3G > VM-3W ≈ VM-3Y. These data indicated that the modification of the charge was more effective than the hydrophobic changes. The higher electrostatic binding between cationic peptide and specific sites on the bacterial membrane may accelerate the interaction [25] and the volume of a bulky amino acid may retard the interaction with membrane. However, the α-helical percentage of VM-3K in a membrane mimetic environment was lower than the template peptide, which indicated that the helicity was not a decisive factor affecting the anti-microbial activity. For the mechanism of the bactericidal activities, all the peptides displayed a high percentage of bacterial membrane permeabilization against *S. aureus* at their high concentrations. While no peptide exhibited more than 50% of membrane permeabilization at their MIC concentration, this indicated that these peptides may penetrate into cytoplasm and induce the dysfunction of the biological processes in bacteria without disrupting the integrity of the cell membrane [26]. This illustrated that the VM family peptides showed multiple mechanisms of action at different concentrations, including bacterial membrane disruption and intracellular dysfunction. Furthermore, VM, VM-3W and VM-3Y induced a sharp increase in membrane permeabilization, which indicated that these peptides had reached a threshold and then destroyed the integrity of bacterial membrane at higher concentration [27]. These peptides may interact with bacterial membranes through electrostatic and hydrophobic interactions, and accumulate until a certain concentration is reached, sufficient to damage the lipid bilayer by acting in a detergent-like manner, as in the carpet or pore formation model [28]. However, VM-3G displayed a concentration-dependent membrane permeabilization that was apparently different with the aforementioned three analogue peptides. VM-3G had already shown significant permeable activity at the low concentration of 1 µM. This may be caused by the fact that glycine is the smallest amino acid, which is amphiphilic and flexible, and its positioning on the N-terminus may help the peptide penetrate into the cell membrane more readily [29]. A study on melittin [30] revealed that, when the concentration of peptide had not yet reached a critical peptide-to-lipid ratio (which was the threshold concentration), the peptide could insert inside the lipid bilayer with the hydrophilic surface of peptide contacting the polar head of lipids and the hydrophobic surface of peptide contacting the hydrocarbon tail of the bilayer to decrease the thickness of and weaken the stability of the membrane structure. In a different situation, the peptide may damage the target cell through perturbing and reorganising the lipid without forming pores [31]. After reaching the threshold concentration, the peptide oriented perpendicular to the membrane and proceeded to pore formation. The simple structure of glycine may help VM-3G more readily enter the interior of the bilayer and thin the membrane to form a pore.

As for the antimicrobial activity against Gram-negative bacteria, it has been noted that only VM-3K had significantly enhanced bactericidal ability against three types of bacteria, which suggested that the increase in cationic character by replacing the third proline with lysine, was effective in enhancing Gram-negative antibacterial effects [32,33].

The VM analogues also showed potent activities against bacterial biofilms. The peptides effectively inhibited the growth of bacterial biofilm, which may be caused by an interference with biofilm maturation. The peptide may interfere with the function of the cell membrane and inhibit the adhesive ability of bacteria in order to decrease cell survival before the formation of biofilm [34]. Most MBIC values of these peptides were twice those of their MIC values. This may be because the sessile bacteria absorb nutrients more easily and have the ability to remove toxic components, and the extracellular polysaccharide (EPS) matrix can protect bacteria from attack [35,36]. Additionally, to ensure the growth of biofilm, the chosen medium may increase the tolerance of bacteria to protect themselves from peptide. It was noteworthy that all peptides displayed eradication abilities against preformed biofilm, which indicated that these peptides have the ability to penetrate into or disrupt the function of the extracellular matrix of biofilm. The peptides may target the extracellular polysaccharides and/or other extracellular polymeric substances to interfere with the function of mature biofilm [37].

With respect to toxicity against erythrocytes, most of the modified peptides appear to be more haemolytic than the template peptide. All four modified peptides showed higher cytolytic activities at low concentrations, which indicated that they were more sensitive to lipid bilayers than was VM. A study on lactarcin showed that replacing the hinge structure with a flexible one (which meant replacing specific amino acids with glycine to break the ‘helix-hinge-helix’ structure) depressed the selectivity to zwitterionic cell membranes and enhanced the cytolysis against eukaryotic cells [38]. So, the replacement of proline may make VM-3K, -3G and -3W lose the hinge structure and enhance sensitivity to erythrocyte membranes. However, considering their significant bactericidal abilities, modified peptides did not display lower selectivity between erythrocytes and pathogens, and the selectivity indices of these three were comparable to template peptide, VM. VM-3K and VM-3Y showed around 10% haemolysis at their 4× MIC, which was acceptable for a therapeutic drug. It has been noted that VM-3Y had a prominent selectivity index and low haemolytic activity. This result may suggest that the increase in the helicity of VM-3Y enhanced the selectivity between erythrocyte and bacterial membranes [39]. However, for VM-3G, which also exhibited an enhancement of helicity, the flexible structure may lose control over of all types of lipid bilayers and the hinge at the third position may be a constrained structure for membrane interaction. Combining the results of the bacterial membrane permeabilization assays, the high flexibility at the inner lipid bilayer of VM-3G, resulted in very limited selectivity between the bacterial and red blood cell membranes. In addition, tyrosine has a bulky side-chain, which may also build hinge at the N-terminus, and the polar functional group of tyrosine differed from the non-polar residue of tryptophan which led VM-3Y to display many differences on cytotoxicity to red blood cells compared with VM-3W, though they both had aromatic side groups [40].

The bioavailability of a peptide is important in clinical applications, and the bacterial infection model using *Galleria mellonella* larvae (waxworms) can evaluate the efficacy of the peptide in vivo [41]. In vivo, VM-3K displayed significant activity in promoting the survival of *S. aureus*-infected waxworms at a much lower concentration compared with template peptide. However, the other three peptides showed less efficiency as anti-bacterial agents in the waxworm, which may relate to their lower stabilities and half-lives [42]. Furthermore, at 4× MICs, all five peptides displayed no toxicity to the waxworms, showing that it was possible to use these as anti-microbial agents, which illustrated the safety of using short peptides such as these in vivo [43]. Interestingly, VM-3W possessed a high haemolytic activity at 4× MIC, but it still showed no toxicity to the waxworm. It was suspected that VM-3W may exhibit low efficiency and stability in vivo, such that much less peptide is available to attack normal cells. Moreover, the environment in vivo is much more complex than that of the haemolytic assay and a cationic and hydrophilic peptide could be adsorbed by plasma or tissue proteins or that the waxworms had a higher tolerance to this peptide [44].

Last but not least, VM and modified peptides did not induce drug resistance after passage in *S. aureus* over 16 times. Traditionally, antibiotics kill bacteria targeting the disruption of a specific structure in the bacterial cell wall or cell membrane or the inhibiting synthesis of proteins and nucleic acids [45]. The overuse of antibiotics-induced bacterial mutants to protect themselves by producing efflux pumps to expel the drugs, modifying their target molecules and decreasing uptake pathways [46]. AMPs act as a part of normal endogenous immunity and have a much broader spectrum of inhibitory activities towards many types of microbes and multiple modes of action to combat the drug-resistance of evolving pathogens [47]. The results generated in this study have illustrated that VM-family peptides have the properties to meet the challenge of bacterial resistance.

## 5. Conclusions

In conclusion, VM, a short-chain α-helical peptide from the venom of the wasp, *Vespa mandarinia*, has potent antibacterial activity against Gram-positive bacteria both in vitro and in vivo, which presumes the multiple mechanisms of bacterial inhibitory actions. After replacing proline at the third position from the N-terminus of VM and synthesising a series of analogues, the antibacterial and antibiofilm activities were found to be enhanced and the variation in the physicochemical properties of amino acids led to different antibacterial mechanisms in the peptides. The haemolytic cytotoxicity of modified peptides was higher than template peptide at working concentrations, but along with the significant biological functions, modified peptides presented comparable selectivity to VM but was even much better for VM-3Y. In vivo, VM-3K showed high stability and bacterial inhibitory efficiency with no toxicity observed in waxworms. The properties of low toxicity, along with broad antimicrobial activity against Gram-positive bacteria, Gram-negative bacteria and yeast and fast killing kinetics and the discouragement of bacterial resistance, suggest that VM-3K could be a potential antimicrobial peptide for consideration as clinical candidate for combating microbial resistance.

## Figures and Tables

**Table 1:** The peptides synthesised and their sequences. The differences of the modified positions in analogues are indicated by underlining.

Peptide Name	Peptide Sequence
VM	FLPIIGKLLSGLL-NH_2_
VM-3K	FLKIIGKLLSGLL-NH_2_
VM-3G	FLGIIGKLLSGLL-NH_2_
VM-3W	FLWIIGKLLSGLL-NH_2_
VM-3Y	FLYIIGKLLSGLL-NH_2_

**Table 2:** The sequences of the peptide VM and its analogues. Hydrophobicity (*H*) was calculated using HeliQuest [21].

Peptide	Hydrophobicity (<*H*>)	Hydrophobic Moment <μM>	Net Charge
VM	1.045	0.618	+1
VM-3K	0.913	0.744	+2
VM-3G	0.989	0.671	+1
VM-3W	1.162	0.508	+1
VM-3Y	1.063	0.601	+1

**Table 3:** α-helical content of five VM peptides calculated by BeStSel [20].

	% of α-Helix in 10 mM NH_4_Ac	% of α-Helix in 50% TFE/NH_4_Ac
VM	0.3	64.3
VM-3K	2.5	60
VM-3G	11.7	75.8
VM-3W	24	63.1
VM-3Y	38.0	75.1

**Table 4:** MICs and MBCs of peptides and analogues against seven microorganisms (µM). Two mg/mL of amphotericin B for *C. albicans* and 2 mg/mL norfloxacin for six strains of Gram-positive and Gram-negative bacteria were used as the positive controls. Data were collected in nine replicates from three independent experiments.

	*S. aureus*	MRSA	*E. faecalis*	*E. coli*	*K. pneumoniae*	*P. aeruginosa*	*C. albicans*
VM	4/4	4/4	16/16	256/256	>512	>512	32/32
VM-3K	1/1	1/2	2/2	8/8	32/32	16/16	32/32
VM-3G	1/1	2/2	4/4	64/128	>512	128/128	>512
VM-3W	1/1	2/2	4/4	>512	>512	>512	>512
VM-3Y	2/2	2/2	4/4	>512	>512	>512	>512

**Table 5:** MBICs (µM) and MBECs (µM) of VM and its four designed analogues against the test bacteria. Data were collected in nine replicates from three independent experiments. ND means not detected.

	*S. aureus*	MRSA	*E. faecalis*	*E. coli*	*K. pneumoniae*	*P. aeruginosa*
VM	8/64	8/64	16>512	>512/>512	>512/>512	>512/>512
VM-3K	2/32	2/64	4/>512	16/>512	64/>512	32/>512
VM-3G	2/32	2/32	8/>512	256/>512	>512/>512	>512/>512
VM-3W	4/128	4/128	16/>512	>512/>512	>512/>512	>512/>512
VM-3Y	2/128	2/64	64/>512	>512/>512	>512/>512	>512/>512
Melittin	4/ND	8/ND	8/ND	32/ND	128/ND	64/ND

**Table 6:** The peptide concentrations that caused 50% lysis of horse red blood cells. (HC_50_, µM).

Peptide Name	HC_50_ (µM)	SI ^1^
VM	44.43	11.11
VM-3K	11.20	11.20
VM-3G	9.686	9.686
VM-3W	9.682	9.682
VM-3Y	211.2	105.6

1: Selectivity index, HC_50_ value/the minimum concentration value of inhibiting microbial growth.

## Data Availability

Not applicable.

## Supplementary Materials

The following supporting information can be downloaded at: https://www.mdpi.com/article/10.3390/biology11091263/s1, Figure S1: The RP-HPLC chromatograms of (a) VM, (b) VM-3K, (c) VM-3G, (d)VM-3W, (e) VM-3Y; Figure S2: MALDI-TOF mass spectra of the corresponding peak in RP-HPLC of (a) VM, (b) VM-3K, (c) VM-3G, (d)VM-3W, (e) VM-3Y.
