# Supplementary material for: Evaluation of the Antimicrobial Properties of a Natural Peptide from Vespa mandarinia Venom and Its Synthetic Analogues as a Possible Route to Defeat Drug-Resistant Microbes
Source: Biology (Basel). 2022 Aug 25;11(9):1263. doi: 10.3390/biology11091263 (PMC9495676; doi:10.3390/biology11091263)
Supplement: Supplementary file 1 [file biology-11-01263-s001.zip › biology-1837295-supplementary.pdf]

Supplementary Material

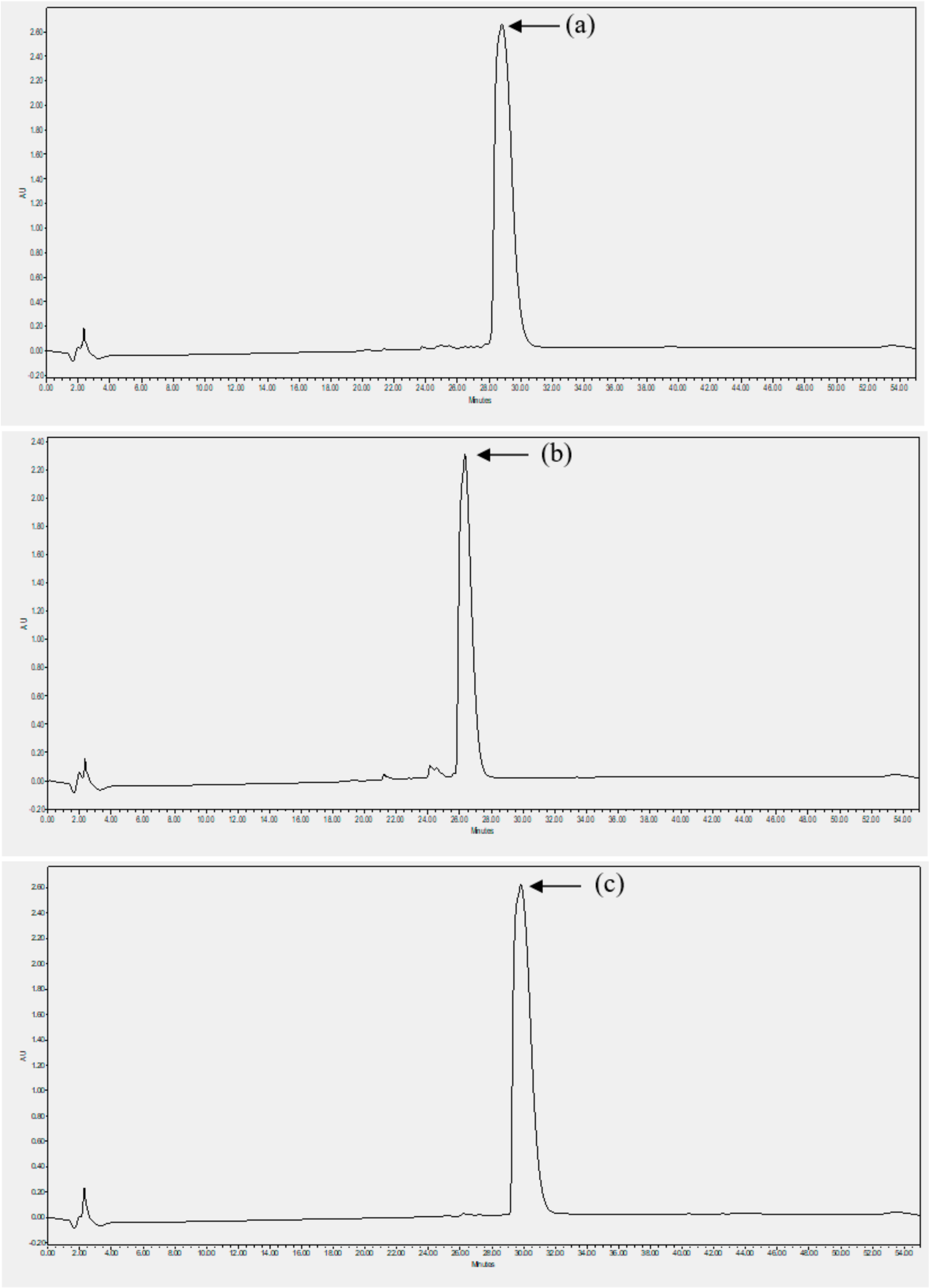

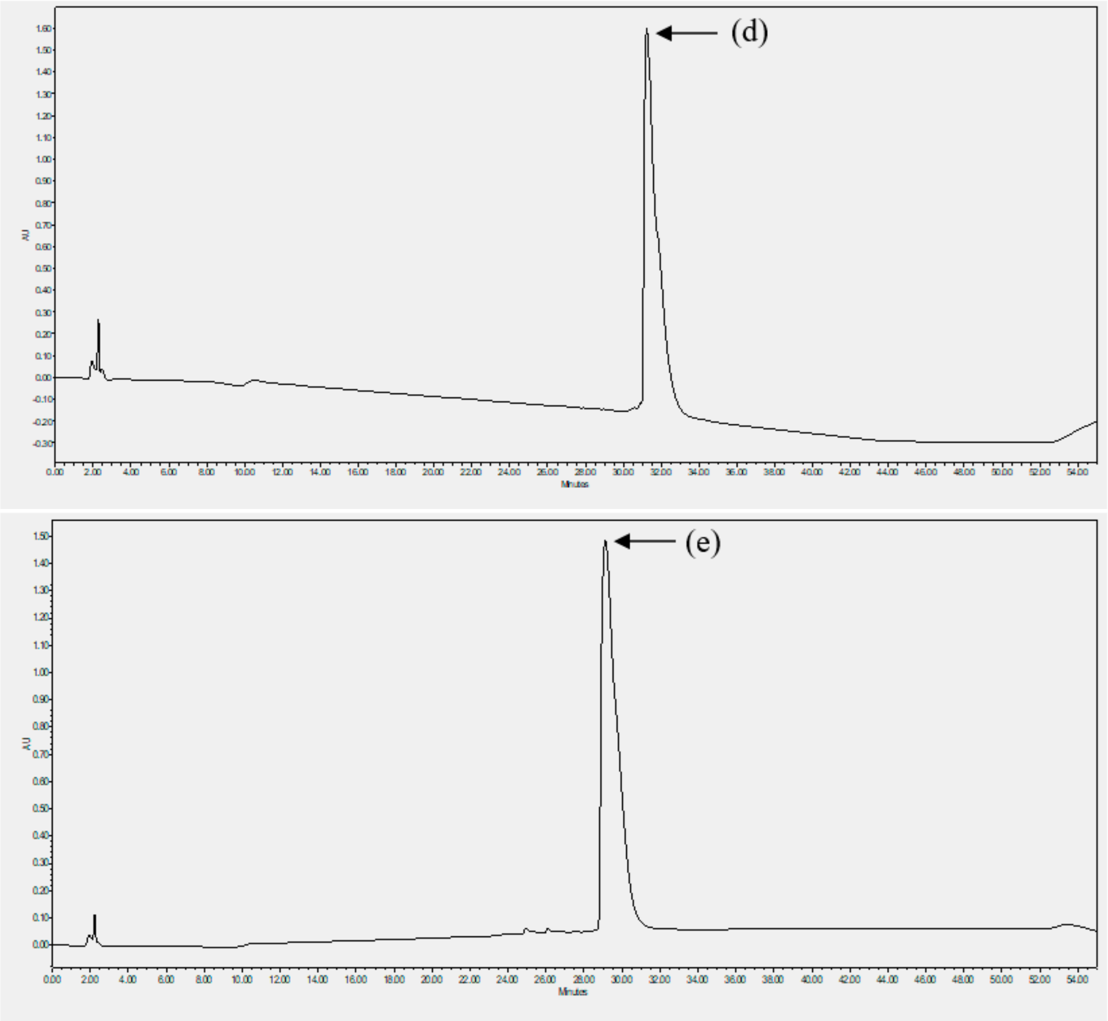

Figure S1. The RP-HPLC chromatograms of (a) VM, (b) VM-3K, (c) VM-3G, (d)VM-3W, (e) VM-3Y. The arrows stand for the elution peak of the corresponding peptides.

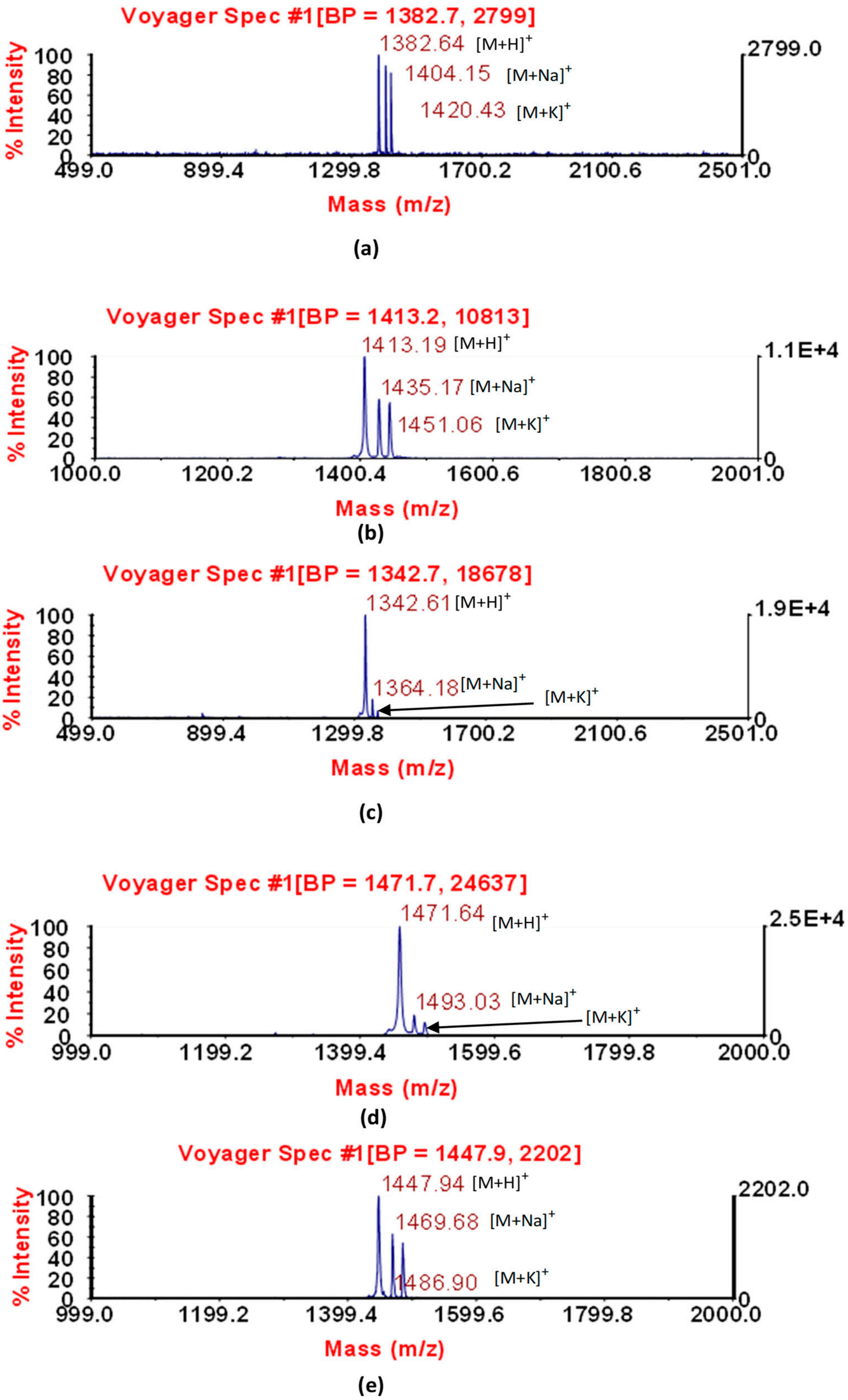

Figure S2. MALDI-TOF mass spectra of the corresponding peak in RP-HPLC of (a) VM, (b) VM-3K, (c) VM-3G, (d)VM-3W, (e) VM-3Y. The peaks corresponding to singly charged protonated ions of the peptide (m/z monoisotopic peaks: $[M+H]^+$, sodium ion adduct: +22 Da, $[M+Na]^+$ and potassium ion adduct: +38 Da, $[M+K]^+$) are indicated.
